# Single-molecule imaging with longer X-ray laser pulses

**DOI:** 10.1107/S2052252515016887

**Published:** 2015-10-21

**Authors:** Andrew V. Martin, Justine K. Corso, Carl Caleman, Nicusor Timneanu, Harry M. Quiney

**Affiliations:** a ARC Centre of Excellence for Advanced Molecular Imaging, School of Physics, University of Melbourne, Parkville, Victoria 3010, Australia; b Department of Physics and Astronomy, Uppsala University, Box 516, SE-751 20 Uppsala, Sweden; c Center for Free-Electron Laser Science, DESY, Notkestrasse 85, DE-22607 Hamburg, Germany; d Department of Cell and Molecular Biology, Uppsala University, Box 596, SE-751 24 Uppsala, Sweden

**Keywords:** coherent diffractive imaging, single-molecule imaging, radiation damage, ‘self-gated’ pulses, XFELs

## Abstract

During the last five years, serial femtosecond crystallography using X-ray laser pulses has been developed into a powerful technique for determining the atomic structures of protein molecules from micrometre- and sub-micrometre-sized crystals. One of the key reasons for this success is the ‘self-gating’ pulse effect, whereby the X-ray laser pulses do not need to outrun all radiation damage processes. Instead, X-ray-induced damage terminates the Bragg diffraction prior to the pulse completing its passage through the sample, as if the Bragg diffraction were generated by a shorter pulse of equal intensity. As a result, serial femtosecond crystallography does not need to be performed with pulses as short as 5–10 fs, but can succeed for pulses 50–100 fs in duration. It is shown here that a similar gating effect applies to single-molecule diffraction with respect to spatially uncorrelated damage processes like ionization and ion diffusion. The effect is clearly seen in calculations of the diffraction contrast, by calculating the diffraction of the average structure separately to the diffraction from statistical fluctuations of the structure due to damage (‘damage noise’). The results suggest that sub-nanometre single-molecule imaging with 30–50 fs pulses, like those produced at currently operating facilities, should not yet be ruled out. The theory presented opens up new experimental avenues to measure the impact of damage on single-particle diffraction, which is needed to test damage models and to identify optimal imaging conditions.

## Abstract 2

A theoretical investigation is presented of how radiation damage gates X-ray laser diffraction from single isolated protein molecules. The impact of this effect on the feasibility of X-ray laser single-molecule imaging with pulse durations of the order of 10 fs is discussed.

## 1. Introduction   

X-ray free-electron laser (XFEL) pulses are envisioned to probe the structures of radiation-sensitive samples, like biological molecules, by outrunning radiation damage processes (Neutze *et al.*, 2000 ▸). However, current facilities produce their brightest pulses with durations of the order of tens of femtoseconds (Emma *et al.*, 2010 ▸; Ishikawa *et al.*, 2012 ▸), which is sufficient time for ionization to become widespread and for ions to move several ångströms (Caleman *et al.*, 2009 ▸, 2011 ▸). In spite of this, the first applications of XFELs to serial crystallography have been highly successful (Chapman *et al.*, 2011 ▸; Boutet *et al.*, 2012 ▸). It turns out that, even for longer pulses (∼ 50–100 fs), Bragg diffraction probes the undamaged structure in the first few femtoseconds of the pulse–sample interaction, turning off at later times when radiation damage distributes the diffraction signal as a diffuse background (Barty *et al.*, 2012 ▸). In this way, XFEL Bragg diffraction is effectively gated by damage, because the expected number of photons scattered to a Bragg peak is equivalent to that produced by a shorter pulse with the same intensity.

Despite the great progress in coherent imaging using XFEL sources, the holy grail – atomic resolution of a single (non-crystalline) biomolecule (Neutze *et al.*, 2000 ▸; Miao *et al.*, 2001 ▸) – has not yet been realised. Nevertheless, the potential reward for success has kept this pursuit at the forefront of research in XFEL imaging science. One of the limiting factors is radiation damage. For non-crystalline samples, diffraction from the undamaged structure is not enhanced by periodicity and is mixed indistinguishably with the diffraction of a damaged structure. This seems to be a major setback from the development of three-dimensional single-particle imaging into a high-resolution technique for single molecules. For example, Hau-Riege *et al.* (2005 ▸) found that radiation damage causes large discrepancies from the ideal diffracted intensities, which led them to conclude that pulses must be no more than a few femtoseconds in duration to avoid severe resolution loss. A more recent study with more detailed scattering models reached a similar conclusion (Ziaja *et al.*, 2012 ▸). However, these studies assessed feasibility with metrics inspired by crystallography whose suitability for single-molecule imaging is disputed (Quiney & Nugent, 2011 ▸). Without accounting in detail for the way that structural information is extracted from single-molecule diffraction data, the issue of damage limits for single-molecule imaging remains inconclusive.

One of the most actively pursued routes to single-molecule imaging involves measuring diffraction from thousands of copies of a molecule one by one. The resulting data are extremely noisy and the molecular orientations are not known. The issue of molecular orientation must be resolved to assemble a three-dimensional data set, which can be performed by several algorithms (Loh & Elser, 2009 ▸; Fung *et al.*, 2009 ▸; Giannakis *et al.*, 2012 ▸; Kassemeyer *et al.*, 2013 ▸; Yefanov & Vartanyants, 2013 ▸). The hallmark of these methods is that they are able to cope with signals as low as 0.01 photons per Shannon–Nyquist pixel (Tegze & Bortel, 2012 ▸). After the three-dimensional data set has been assembled, the atomic structure is recovered *via* coherent diffractive imaging (CDI) methods (Miao *et al.*, 1999 ▸; Marchesini, 2007 ▸). Since the first demonstration of CDI with an X-ray synchrotron source around 15 years ago (Miao *et al.*, 1999 ▸), a rich array of CDI techniques has been developed for applications to biology and materials science (Miao *et al.*, 2015 ▸).

**Figure 1:**
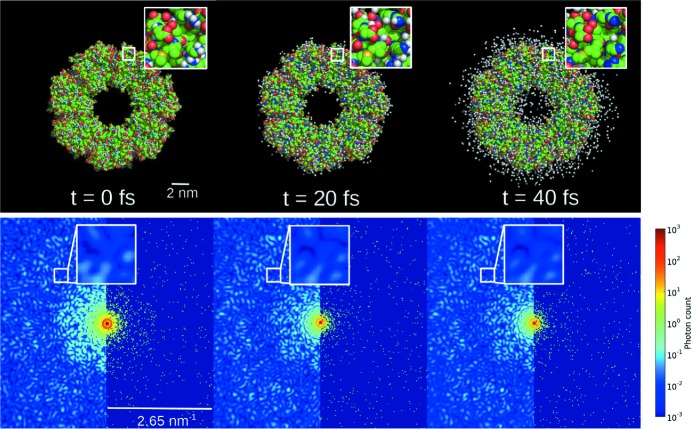
A graphical representation of ion diffusion in GroEL, where ion locations are chosen stochastically using the time-dependent temperature. Simulation parameters are: 8 keV, 5.0 × 10^20^ W cm^−2^ and 100 nm pulse diameter. Ionized hydrogen (white) moves much faster than ions of other elements. The accumulated diffraction pattern for each pulse time is shown below and these were generated by randomly assigning each atom an ionization state and a displacement according to the rate-equations model described in Appendix *A* [App appa]. The effect of shot noise is shown on the right-hand half of each diffraction image. Intensities are normalized with the same number of incident photons (10^12^) prior to calculating shot noise. There are correlations between the speckle structure at early and late pulse times due to the gating effect, as shown by the inset regions.

The crucial information needed to resolve the unknown orientations and, ultimately, the structure is contained in the modulations of the diffraction signal arising from interference between different atoms, often called ‘speckles’ (see Fig. 1 ▸). Radiation damage changes the structure of the sample dynamically, such that the final diffraction pattern is the sum of the diffraction from many modified structures, each with a different distribution of ions and ion displacements. It has been shown that averaging the diffraction over different molecular configurations (Maia *et al.*, 2009 ▸) lowers the speckle contrast relative to the mean scattering intensity within each resolution shell. We expect radiation damage to cause a similar loss of contrast. Not only is the amplitude of the speckle structure reduced, but the speckle structure also fluctuates from shot-to-shot due to damage, in addition to the fluctuations due to changing orientation and shot noise. We will use the term ‘damage noise’ to refer to these fluctuations of the speckle structure due to damage. So far, damage noise has not been considered in studies of three-dimensional data assembly. Here, we present calculations of damage noise per diffraction pattern due to spatially uncorrelated damage processes, which include ionization and ion diffusion but not the Coulomb explosion of the molecule. An analysis of damage noise as a function of pulse duration reveals a gating effect in single-molecule diffraction, whereby long pulses measure an equivalent amount of information about the average structure to shorter pulses of the same intensity. Theoretical predictions of damage noise are also the first step to understanding how orientation determination and three-dimensional data assembly can be performed with data affected by radiation damage.

It is important to clarify how the gating effect for single-molecule diffraction compares with the case of crystal diffraction. In the gating effect for crystal diffraction, the Bragg peaks accumulate intensity until a resolution-dependent cutoff time determined by damage, while a diffuse background continues to increase until the pulse has left the sample while gradually degrading the signal-to-noise ratio. In single-molecule diffraction, the initial part of the pulse produces a speckle pattern that encodes the initial ion positions, up to a cutoff time determined by damage. The speckle amplitude derived from early pulse times thus carries analogous information to the Bragg scattering from crystals. As with crystals, the diffraction from the damaged sample by the tail of the pulse continues to scatter a diffuse speckled pattern. Without the coherent amplification of a crystal, the pristine signal from early pulse times will be of a similar order of magnitude to the diffraction from the damaged molecule at later pulse times. If the damage processes are uncorrelated between measurements, so that diffraction from the damaged molecule at later pulse times is noise-like, then it will contribute a featureless background to the merged three-dimensional intensity. In these circumstances, the speckle structure of the merged three-dimensional intensity will still encode structural information about the initial ion positions, and the amplitude of the merged speckle structure will be relatively insensitive to pulse duration due to the gating effect.

An alternative to alignment *via* post-processing is experimentally to align isolated gas-phase molecules, using, for example, quantum-state selection methods (Küpper *et al.*, 2014 ▸; Stern *et al.*, 2014 ▸). A great advantage of this approach is that multiple molecules can be illuminated simultaneously, increasing the signal-to-noise ratio and, as supported by the work here, reducing the impact of damage. These methods have been demonstrated only for small (2,5-diiodo­benzo­nitrile) molecules so far (Küpper *et al.*, 2014 ▸; Stern *et al.*, 2014 ▸) and extensions to larger molecules are being actively pursued. If the molecules are aligned experimentally, the self-gating effect still applies. Radiation damage modifies each molecule in the beam uniquely and stochastically, so that multiple damage scenarios are averaged in a single diffraction measurement in an analogous way to crystallography. This increases the signal with respect to both damage noise and shot noise. The self-gating effect ensures that the benefits of using multiple aligned molecules are not lost entirely by using X-ray pulses longer than 10 fs.

Once the three-dimensional data assembly has been performed, damage will still have a residual effect on the resulting three-dimensional diffraction volume. Damage reduces the contrast in the averaged diffraction volume (Quiney & Nugent, 2011 ▸) and, depending on the theoretical perspective, also contributes a background (Lorenz *et al.*, 2012 ▸). Promisingly, the reduction in contrast can be accounted for during structure determination by treating the sample in terms of a small number of structural modes (Quiney & Nugent, 2011 ▸). The background contribution is expected to be small for hard X-rays at the beam conditions currently available.

In addition to analysing the damage noise, we show how the mean and standard deviation of the diffraction signal can be combined into a sensitive measure of damage. An advantage of the measure we propose is its sensitivity to both ionization and ion motion, whereas the mean signal alone depends only on ionization. There is a need to measure damage experimentally and provide some validation and clarification for theoretical damage modelling. Many different types of damage model have been developed, based on rate equations (Hau-Riege *et al.*, 2004 ▸), molecular dynamics (Neutze *et al.*, 2000 ▸; Jurek *et al.*, 2004 ▸) or plasma theory (Caleman *et al.*, 2009 ▸), and each has specific advantages and disadvantages. For example, molecular dynamics models can keep track of specific ion trajectories, but are only computationally tractable for small molecules (Neutze *et al.*, 2000 ▸). Rate-equations models can simulate damage to large molecules but ignore information about ion motion on atomic length scales (Hau-Riege *et al.*, 2004 ▸). Experimental measurements of damage will provide valuable feedback on our theoretical understanding of the interaction between XFEL pulses and biomolecules, which is needed to develop single-molecule imaging techniques.

## 2. The effect of radiation damage on diffraction contrast   

The goal of single-molecule imaging is to recover the initial position **R** of each atom in the sample. For simplicity, we will give equations for the case of a single atomic species, noting that the generalization to multiple atomic species for all key results is given in Appendix *E* [App appe] and is similar to that found in Quiney & Nugent (2011 ▸). The intensity of a single measurement of a single molecule can be written
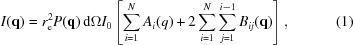
where **q** is the scattering vector with magnitude *q*, dΩ is the solid-angle term, *r*_e_ is the classical electron radius, *N* is the number of atoms and *P*(**q**) is a polarization term that will be ignored in this discussion. To simplify the mathematical notation, we assume that the incident intensity takes a uniform value *I*_0_ for the duration of the pulse. We define
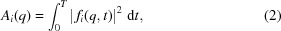
and
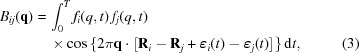
where 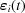 is the displacement of the *i*-th atom from its initial position and *T* is the duration of the pulse. For a single two-dimensional measurement, it is understood that **q** is sampled at points on the Ewald sphere, but in general we will use **q** to be a general three-dimensional vector and *I*(**q**) is a three-dimensional function. The atomic scattering factor *f*(*q*,*t*) depends upon the ionization state of the atom, which changes as a function of time. The ionic scattering factors can be calculated using Slater orbitals (Slater, 1930 ▸) and we use *f*_0_(*q*) to denote the atomic scattering factor of the unionized atom. We assume that the probability of an ion having a particular ionization state at time *t* is independent of where that atom is located in the sample. Although the ionization state as a function of time is different for each atom, statistically atoms of the same atomic species are assumed to be equivalent. We write *A*(*q*) and *B*(*q*) as functions of the magnitude of the scattering vector, *q*, because we assume the atomic scattering factors are spherically symmetric.

Consider an ensemble of two-dimensional diffraction measurements, each with a unique damage scenario. For three-dimensional imaging, the data need to be assembled into a three-dimensional intensity volume using an algorithm that accounts for the unknown molecular orientations. The desired solution of the algorithm is an average intensity, where each two-dimensional measurement is correctly placed according to orientation and the different damage scenarios are averaged. As shown in Appendix *B* [App appb], the average intensity can be written in the form
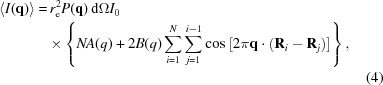
where we have
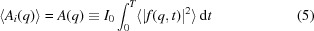
and
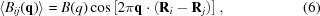
where
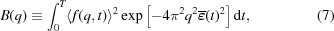
and 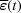 is the root mean square (r.m.s.) displacement of an ion as a function of time.

If the analysis is restricted to damage processes that are random and spatially uncorrelated, then we can treat the terms *A_i_*(*q*) and *B_ij_*(**q**) as random variables and study the effect of damage statistically. We also treat the initial atomic positions **R**_*i*_ as random with a uniform probability distribution, as is done in crystallography to analyse the statistics of Bragg intensities (Wilson statistics) at high scattering angles (*q* > 0.33 nm^−1^) (Huldt *et al.*, 2003 ▸). Both ionization and ion diffusion can be treated within this framework and, as we will show, both are involved in a self-gating pulse effect. Expansion of the molecule by Coulomb forces is not covered by the statistical treatment presented here, but is discussed further below.

The second term on the right-hand side of equation (4) is sensitive to the atomic positions and accounts for the contrast in the average diffraction pattern. We can treat this information as the ‘signal’ we aim to measure. The contribution each atom makes to the signal is proportional to *B*(*q*), which is equal to the standard deviation of the diffraction in the merged three-dimensional data set divided by the number of atoms. The mean shot noise level, denoted by σ_*N*_, is proportional to the square root of the intensity. We can estimate the mean shot noise level by considering the mean diffracted intensity in a shell of constant *q*, which can be derived by integrating equation (4) and is proportional to *A*(*q*). When the signal is compared with the noise, the proportionality constants have no influence on the interpretation, so we drop them for simplicity and write
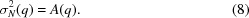
In addition to shot noise, there is also the damage noise due to the variations in how the damage manifests in each measurement. One contribution to the damage noise is the fluctuation of *A_i_*(*q*), which is characterized by the standard deviation of *A_i_*(*q*), which we denote by σ_*A*_(*q*). The second contribution to damage noise is the deviation of *B_ij_*(**q**) from the average speckle *B*(**q**), which has a standard deviation σ_*B*_(*q*). The term σ_*B*_(*q*) is given by the difference between the standard deviation of the second term on the right-hand side of equation (1) minus the standard deviation of the second term on the right-hand side of equation (4). In Appendices *C* [App appc] and *D* [App appd], we provide derivations of σ_*A*_(*q*) and σ_*B*_(*q*) that give the following results:
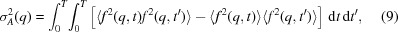
and
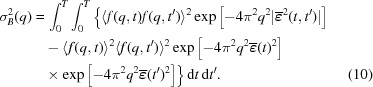
By comparing the size of the signal with the size of the shot noise and damage noise levels (subscript *ND*), we can gauge how much information on the molecule’s structure is contained in each measurement. Here, we will study how the diffraction pattern varies as a function of pulse duration and pulse energy. We propose the following signal-to-noise ratio (SNR) to characterize the diffraction
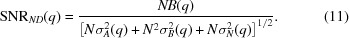
It is also interesting to compare the signal directly with the damage noise (subscript *D*), ignoring shot noise, using the following ratio
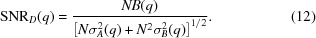
To estimate SNR_*ND*_(*q*) and SNR_*D*_(*q*), we need to calculate the statistical averages of the scattering factor, including 〈*f*(*q*, *t*)〉 and 〈*f* ^2^(*q*, *t*)〉, which in turn depend on the expected number of ions in each ionization state as a function of time. To calculate *B*(*q*) and σ_*B*_(*q*), we also need to know the ion temperature as a function of time. These parameters can be calculated by many of the damage models reported in the literature so far, such as molecular dynamics models (Neutze *et al.*, 2000 ▸; Jurek *et al.*, 2004 ▸) and hydrodynamic (rate-equations) models (Hau-Riege *et al.*, 2004 ▸; Scott, 2001 ▸; Caleman *et al.*, 2011 ▸). Here, we will present the results of a rate-equations model to investigate single-molecule diffraction contrast and to explore the extent to which there is a self-gating pulse effect in single-molecule diffraction.

The term that has not been calculated before is the correlation between the scattering factor at different time points, *e.g.* 〈*f*(*q*, *t*) *f*(*q*, *t*′)〉, which is needed to calculate the damage noise levels. To calculate these correlations we need to know the conditional probability *P*[*f*_*n*_(*q*, *t*′) | *f*_*m*_(*q*, *t*)], which gives the probability of an ion being found in ionization state *n* at time *t*′ given that it was in ionization state *m* at time *t*. We have developed a way of calculating these conditional probabilities, and hence the damage noise. First the damage simulation is carried out, generating the populations of ion states at all time points, and the transition rates between ion states are stored as a function of time. Starting with the mean ion population of state *m* at time *t*, the stored transition rates can be used to generate the fraction of these atoms in ionization state *n* at all later time points *t*′ > *t*, from which the conditional probabilities can be readily inferred.

We use a damage model based on a rate-equations model (Hau-Riege *et al.*, 2004 ▸), which is extended to include ion diffusion using the methods from a non-local thermal equilibrium plasma model (Scott, 2001 ▸; Caleman *et al.*, 2011 ▸). The details of the model are given in Appendix *A* [App appa]. As we closely follow the methods of Hau-Riege *et al.* (2004 ▸) and Caleman *et al.* (2011 ▸), we expect the results and the validity of our model to be similar. As we will show, there are sufficient physical processes in our model to illustrate the self-gating pulse effect in single-molecule diffraction.

All statistical quantities are given as weighted averages over the light elements (H, C, N, O), as described in Appendix *E* [App appe]. Sulfur was included in the rate-equations model of damage, but was excluded from the average of statistical diffraction quantities, like *A*(*q*), *B*(*q*) and σ_*B*_(*q*), because it is computationally intensive. Sulfur has a much larger number of possible electron configurations, and averages that depend on two time variables [*e.g.* σ_*B*_(*q*)] took too long to compute for the range of beam conditions we study here. Since there are of the order of 100 S atoms in the molecule studied here and 10^4^ light atoms, our main conclusions are not expected to be affected by neglecting the diffraction from sulfur. The inclusion of sulfur is not problematic for performing single calculations to compare with a specific set of experimental conditions. The approach can be extended to heavier elements for future theoretical studies using parallelization and high-performance computing facilities.

We have set up our simulations using the chemical composition and size of the protein GroEL (PDB code 1grl). It contains a total of 5278 H, 15043 C, 4067 N, 4767 O and 119 S atoms and has an approximate average diameter of 15 nm. This chaperonin molecule is a candidate for first tests of single-molecule imaging because it survives intact in mass spectrometry experiments (Rostrom & Robinson, 1999 ▸), which subject the molecule to similar conditions to injection at an XFEL. It is also of sufficient size to scatter around 10^4^ photons per diffraction pattern, as shown in Fig. 1 ▸.

**Figure 2:**
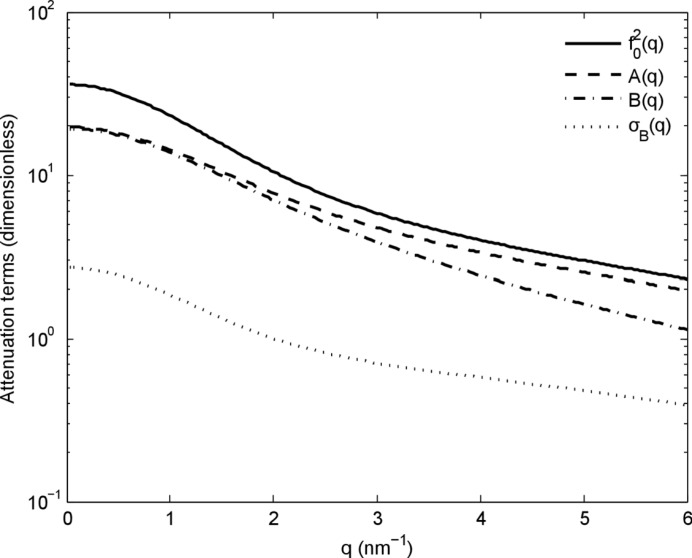
The effects of damage on the atomic structure factor. The term *f*_0_(*q*) is the undamaged atomic scattering factor for an unionized C atom, *A*(*q*) is proportional to the mean intensity per C atom at each resolution shell, *B*(*q*) is proportional to the speckle contrast for carbon and σ_*B*_(*q*) is the standard deviation of the shot-to-shot fluctuations of the speckle due to damage. When there is no damage, *A*(*q*) and *B*(*q*) are equal to 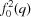. The simulation parameters were 8 keV photon energy, 40 fs pulse duration, 2 mJ pulse energy and spot size of 100 × 100 nm.

Simulations were performed at a photon energy of 8 keV (wavelength ∼0.155 nm), which is sufficient resolution for structural biology and similar to that demonstrated in simulation studies of single-molecule imaging (Tegze & Bortel, 2012 ▸). The principal effects of damage on molecular diffraction can be seen in Fig. 2 ▸, which shows a simulation for a pulse duration of 40 fs, a beam intensity of 5 × 10^20^ W cm^−2^ (corresponding to a 2 mJ pulse) and a spot size of 100 × 100 nm^2^. As the energy bandwidth of an XFEL is typically around 0.2%, we expect it to have no significant impact on damage. Without damage *A*(*q*) would be equal to 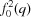, but with damage it is reduced, attenuating the mean intensity by the same amount. The attenuation occurs at all resolutions, but is a greater fraction of the original signal at lower resolutions. As shown in a recent damage study (Caleman *et al.*, 2015 ▸), this effect is due to valence-shell ionization, because the scattering factors of valence electrons scatter at lower angles compared with core–shell ionization or ion diffusion. Ion diffusion attenuates preferentially at higher resolution before lower resolution, and core–shell ionization attenuates both high and low resolution at similar rates. The term *B*(*q*) is lower than *A*(*q*) because of the effects of ion motion and the discrepancy is more pronounced at higher resolution. At 40 fs, the root mean-square (r.m.s.) displacement of ions due to diffusion is around 11 Å for H, less than 0.1 Å for S and 0.2–0.4 Å for C, N and O. This indicates that ion diffusion is not a dominant process under these pulse conditions for the non-H atoms that contribute the bulk of the scattering. The deviations between *A*(*q*) and *B*(*q*) are important for accurate structure retrieval methods (Quiney & Nugent, 2011 ▸). In this case, the most significant damage noise term σ_*B*_(*q*) is lower than *B*(*q*) across all resolutions, indicating that, even for pulse durations as long as 40 fs, damage noise does not exceed the signal from the average molecular structure.

**Figure 3:**
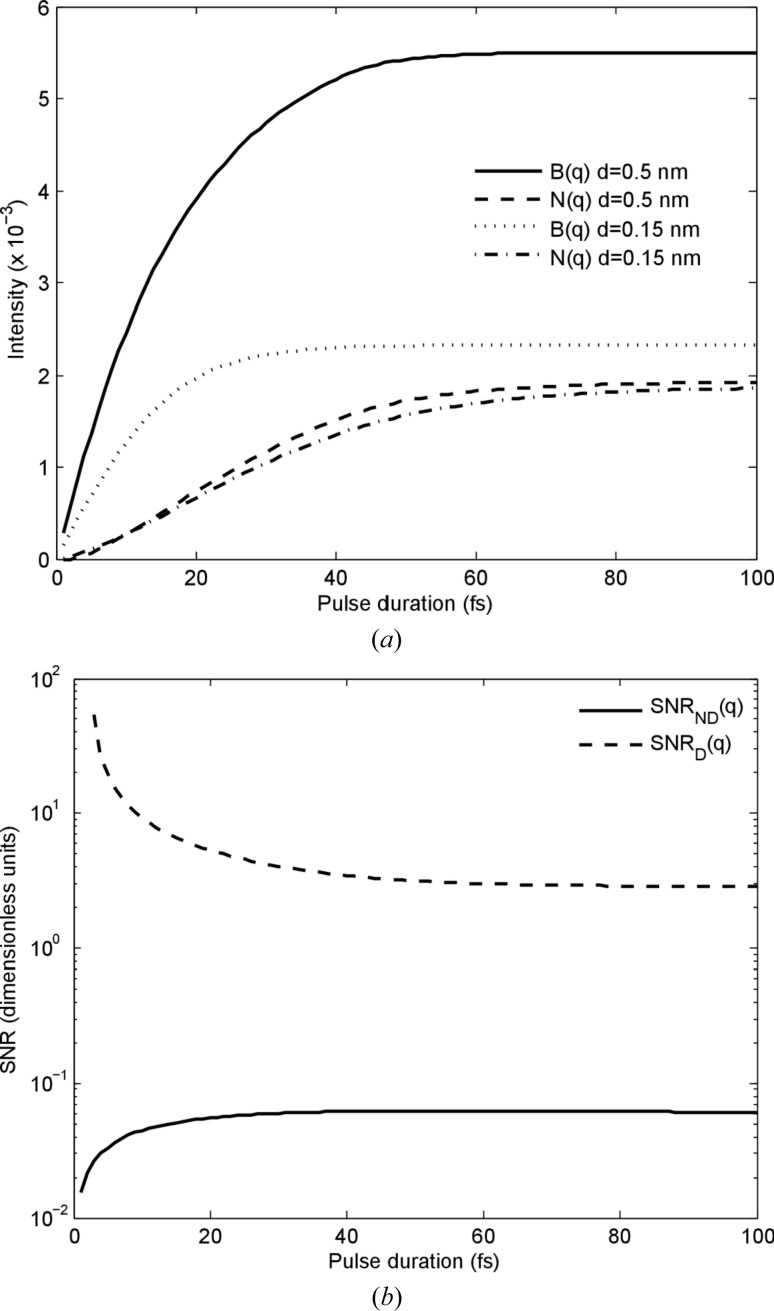
(*a*) Scattering and noise levels (due to damage only) as a function of pulse duration for constant incident intensity (5 × 10^20^ W cm^−2^) at 8 keV photon energy and 100 × 100 nm spot size. *B*(*q*) is proportional to the speckle contrast and we define *N*(*q*) ≡ 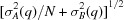, which is the denominator in equation (12) and measures the average contribution to the damage noise per atom. (*b*) Signal-to-noise ratios with and without shot noise for a resolution of 0.15 nm.

To illustrate the self-gating pulse effect in single-molecule diffraction, we plot *B*(*q*) as a function of pulse duration for a constant photon energy (8 keV) and constant beam intensity (5 × 10^20^ W cm^−2^). We see in Fig. 3 ▸(*a*) that the signal level at 0.15 nm resolution rises steadily until it plateaus at a maximum value of around 20 fs. The signal at lower resolution accumulates for longer pulse times. Interestingly, the noise due to radiation damage also rises nonlinearly, accumulating at a slower rate at longer pulse times. This is because the random distribution of ions in the sample has a smaller variation when the bound electrons are almost entirely depleted from each ion. The signal-to-noise ratios, shown in Fig. 3 ▸(*b*), show strikingly that shot noise has a much greater effect than damage noise. Although SNR_*D*_(*q*) improves greatly for short pulses (< 5 fs), SNR_*ND*_(*q*) maximizes when the signal *B*(*q*) maximizes at around 20 fs.

**Figure 4:**
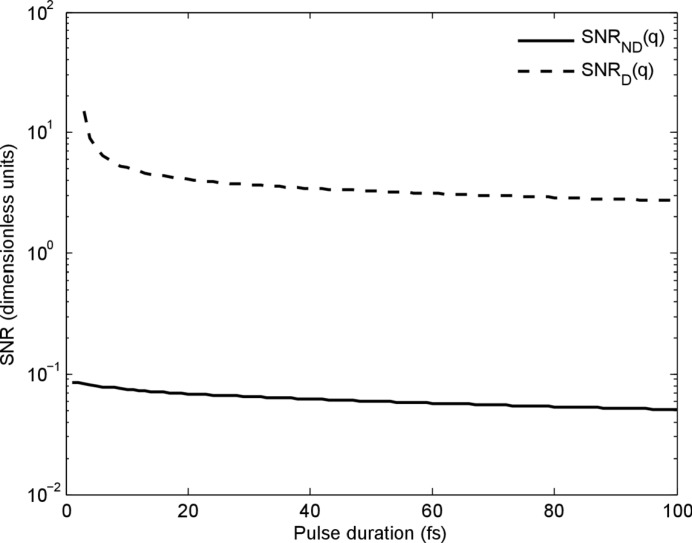
Maximum signal-to-noise ratios with and without shot noise for a resolution of 0.15 nm for 8 keV photon energy, 100 × 100 nm spot size and constant pulse energy of 2 mJ.

The results are interesting when there is an experimental trade-off between pulse duration and pulse energy. For example, the Linac Coherent Light Source (LCLS, Menlo Park, California, USA) can produce 2 mJ pulses with pulse durations of 30–50 fs for hard X-rays (Emma *et al.*, 2010 ▸). Pulses shorter than 5 fs can be produced by the LCLS using a low-charge method or a slotted-foil method, but at the expense of around a factor of ten in pulse energy. Given such a choice, the analysis presented here suggests that the gain in signal from a longer pulse with higher pulse energy compensates for the increase in damage. We note, though, that this conclusion only applies to spatially uncorrelated damage processes like ionization and ion diffusion (not a Coulomb explosion). Fig. 4 ▸ shows that SNR_*ND*_(*q*) and SNR_*D*_(*q*) have a weak dependence on pulse duration at constant pulse energy. This suggests that maximizing pulse energy has a greater influence on the success of single-molecule imaging than pulse duration with respect to the spatially uncorrelated damage mechanisms considered here.

If multiple molecules were simultaneously aligned and exposed to the X-ray pulse (as described in the *Introduction*), we would still expect a gating effect qualitatively similar to that shown in Fig. 2 ▸. However, we would expect SNR_*ND*_(*q*) and SNR_*D*_(*q*) to scale as (*N*_mol_)^1/2^, where *N*_mol_ is the average number of molecules in the beam for each exposure. This is because the signal is proportional to *N*_mol_, while standard deviations of the damage noise and shot noise scale as (*N*_mol_)^1/2^. This analysis lacks the additional fluctuations due to coherent interference between molecules, which have been considered in the context of angular correlation methods (Kirian, 2012 ▸).

## 3. A method of measuring damage experimentally   

**Figure 5:**
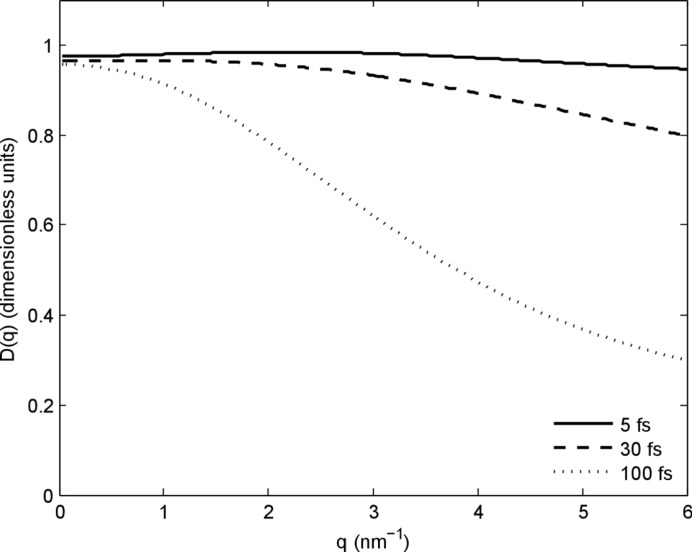
The function *D*(*q*) for different pulse durations for 8 keV photon energy, 100 × 100 nm spot size and constant pulse energy of 2 mJ.

The statistical analysis of diffraction contrast can be used to measure the amount of damage in single-molecule experiments. The average change to the atomic structure factors, characterized by *A*(*q*), can readily be measured by summing diffraction patterns. This provides some information about ionization levels but not ion motion. There is more information to be gained by analysing the fluctuations of the diffraction signal. It is not convenient to measure SNR_*ND*_(*q*), because *B*(*q*) cannot be measured directly without resolving the issue of unknown orientations and assembling a three-dimensional data set, effectively accomplishing a full imaging experiment. An experimentally simpler proposition, which is independent of the imaging experiment, is to measure the standard deviation of the signal within each resolution ring, averaged over all of the measured diffraction patterns. The standard deviation is proportional to 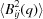 and is a measure of the speckle contrast. It will contain contributions from both the average structure of the sample and the damage noise. Unfortunately it is not clear how to separate those two contributions experimentally. Nevertheless, the standard deviation is a sensitive measure of any dynamic change in the sample structure because it will drop relative to the mean scattering signal, as has been shown for averages of molecular conformation (Maia *et al.*, 2009 ▸). To isolate the effect of damage-induced structural change, we create a measure that first subtracts the expected contribution of shot noise, which is equal to μ_pix_(*q*), and then normalizes by the mean intensity as follows
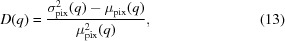
where μ_pix_(*q*) is the average intensity at a pixel in resolution ring *q* averaged over the whole data set and σ_pix_(*q*) is the corresponding standard deviation. The mean and standard deviation are calculated from the ensemble of experimental data of molecules measured individually in random orientations. It possible to show that
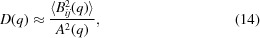
where 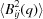 is given in Appendix *D* [App appd]. It is possible to show that 0 < *D*(*q*) < 1, because 〈*f*(*q*, *t*)*f*(*q*, *t*′)〉^2^ < 〈*f* ^2^(*q*, *t*)〉 〈*f* ^2^(*q*, *t*′)〉. Fig. 5 ▸ shows *D*(*q*) for variations in pulse duration at constant pulse energy (2 mJ). The large variations at high scattering angle indicate the sensitivity of *D*(*q*) to ion motion and inner shell ionization, thereby providing complementary information to a measurement of *A*(*q*). The term *D*(*q*) provides a new means of comparing damage simulations with experiment, and testing the assumptions that underpin damage models for the single-molecule case.

For low diffraction intensities, the dominant error in the calculation of *D*(*q*) from experimental data is the error in μ_pix_(*q*), given by
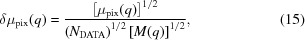
where *N*_DATA_ is the number of diffraction patterns recorded. The term *M*(*q*) is the number of speckles in resolution ring *q*, which is estimated by dividing the circumference of the ring by the expected speckle width 1/*d*, where *d* is the width of the molecule. Assuming *D*(*q*) is of the order of 1, the error in *D*(*q*) varies as δ*D*(*q*) ≃ |δμ_pix_(*q*)|/|μ_pix_(*q*)|. For the test molecule quoted above and a photon energy of 8 keV, a pulse energy of 2 mJ and a spot size of 100 × 100 nm at a resolution of *q* = 6.67 nm^−1^, an accuracy of δ*D*(*q*) = 0.01 can be achieved in of the order of 10^3^ patterns, which is at least an order of magnitude less than the number required to achieve the same resolution in a three-dimensional imaging experiment (Tegze & Bortel, 2012 ▸). This analysis could be used to gain early feedback about the data used in an imaging experiment.

## 4. Expansion of the molecule   

It is predicted from both molecular dynamics (Neutze *et al.*, 2000 ▸) and hydrodynamics simulations (Hau-Riege *et al.*, 2004 ▸) that ions will move due to electrostatic forces for pulse durations longer than 10 fs. This process is sometimes referred to as a Coulomb explosion. The Coulomb explosion is one example of a spatially dependent damage process, as ions at the surface are predicted to move first, while ions near the centre are shielded by the trapped electrons and remain relatively stationary. Unlike the spatially uncorrelated damage processes considered above, the effect of the explosion on the diffraction signal is not readily analysed in terms of signal and noise. Instead, we are in a regime of imaging a dynamic sample. This has been achieved in coherent imaging techniques by modelling the diffraction as a series of modes (Quiney & Nugent, 2011 ▸; Lorenz *et al.*, 2012 ▸; Thibault & Menzel, 2013 ▸) rather than as a single coherent wave, which is the classic assumption underpinning traditional coherent imaging methods. In modal form, the diffracted intensity is written in the form
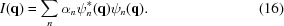
When imaging a dynamic sample, the modes ψ_*n*_(**q**) represent the dominant structural correlations that arise during the dynamic evolution of the sample and α_*n*_ is the corresponding weight. For three-dimensional single-molecule imaging, it is already known that a modal description will be needed to describe the variation in ionization rates between different elements (Quiney & Nugent, 2011 ▸; Lorenz *et al.*, 2012 ▸). Although a full reconstruction accounting for expansion is beyond the scope of this work, we will explore the issues surrounding a modal description of the expansion of a bare molecule and also make some remarks about how this changes if there is a buffer layer around the molecule.

**Figure 6:**
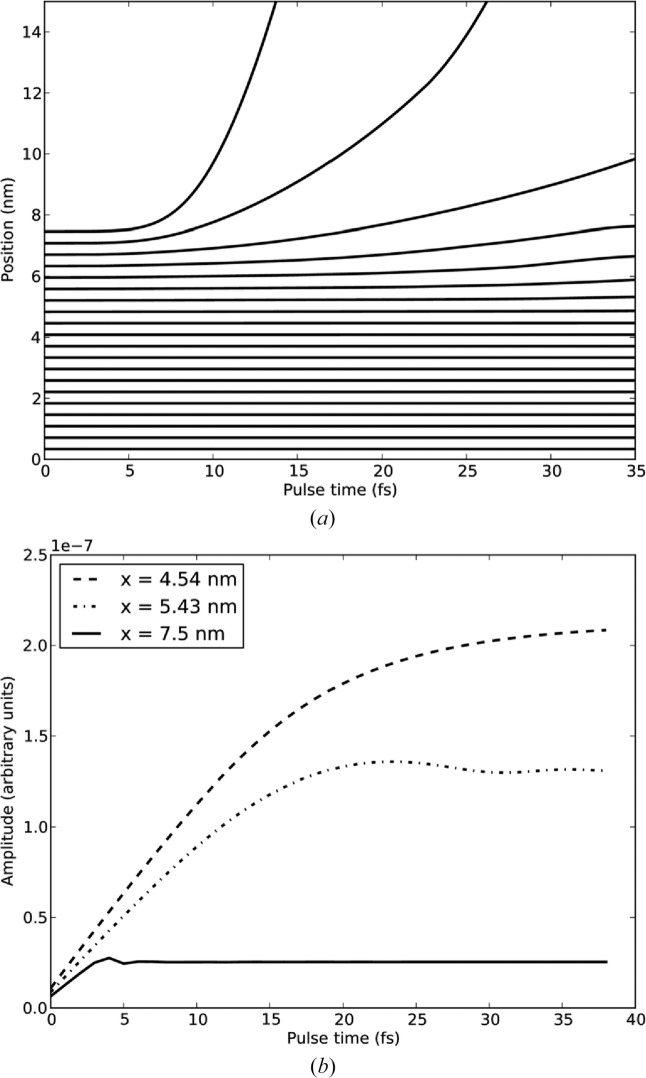
(*a*) The radial position of layers as a function of pulse time. 20 out of 100 layers are shown. (*b*) The amplitude of the interference term for a stationary atom and atoms with initial positions (*x*) of 4.5, 5.4 and 7.5 nm, evaluated at 1.5 Å resolution.

We have performed a hydrodynamic simulation of the expansion of one-dimensional radial layers of the sample, following the methods developed by Hau-Riege *et al.* (2004 ▸) and detailed in Appendix *A* [App appa]. The simulation includes the forces due to ions and trapped electrons and the radiation pressure of the trapped electrons. The radial distribution of trapped electrons is calculated in order to estimate the forces. Fig. 6 ▸ shows the movement of layers for a spherical mol­ecule with the same chemical composition and approximate size (7.5 nm radius) as GroEL for a 40 fs pulse at 8 keV. The model shows the shielding of the inner part of the molecule by the trapped electrons, and those ions within 0.7 of the initial radius move less than 3 Å, but this only accounts for 37% of the ions. The outer layers, which are poorly shielded by trapped electrons, start to move at 10 fs and contain the remaining 63% of ions that move more than 3 Å. These qualitative features are in good agreement with the results of Hau-Riege *et al.* (2004 ▸).

To give an indication of how radial expansion affects the diffraction, we analyse the interference between a C atom in an outer layer with a time-dependent position *r*(*t*) and a C atom at the centre of the molecule that is assumed to be stationary at position *r*_0_ for the duration of the pulse. When the scattering vector and the radial motion direction are parallel, the expectation value of the interference term between these two atoms is given by
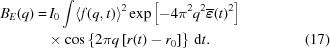
We can evaluate *B*_*E*_(*q*) by setting *r*(*t*) to be the radial motion predicted by the one-dimensional layer simulation. Since *B*_*E*_(*q*) is an oscillatory function of *q*, we evaluate the amplitude of the fluctuations over a range of *q* values as follows
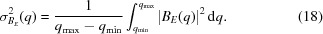
In Fig. 6 ▸(*b*), we plot 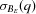 for *q* = 5.7 nm^−1^ (*q*_min_ = 5.4 nm^−1^, *q*_max_ = 6.0 nm^−1^) as function of pulse duration at constant intensity for ions in three different shells. The ion with initial position *x* = 4.5 nm does not move appreciably during 40 fs and the accumulation of 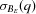 is the same as that of *B*(*q*) in Fig. 5 ▸(*a*) up to an overall scaling. The initial ion position *x* = 5.4 nm moves by about 3 Å by 40 fs and we see that the accumulation of 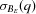 slows as the ion starts to move at around 15 fs. In this case, 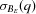 is not monotonic but fluctuates when the radial displacement of the ion approaches 1.5 Å (∼ 1/*q*), and these oscillations dampen as the radial displacement exceeds 1.5 Å. For the ion in the outermost layer (*x* = 7.5 nm), 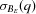 reaches its asymptotic value much sooner at 6 fs, consistent with the very rapid acceleration of the ions closest to the surface.

The tentative conclusion that we can draw from this analysis is that a surface ion only contributes to the contrast of the merged three-dimensional intensity up until the time that it starts to move appreciably. Once it does move, it effectively becomes an incoherent source, at least for high-angle diffraction (*i.e.* length scales smaller than the total displacement of the ion during the pulse). The accumulation of 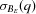 during the pulse resembles the gating effect for spatially uncorrelated damage processes, except that now the cutoff time (at which point the amplitude stops increasing) depends on both the radial position of the ion and the magnitude of the scattering vector, *i.e.* *t*_*c*_(*q*, *R*). In contrast, the gating effect for spatially uncorrelated sources only depends on the magnitude of the scattering vector *t*_*c*_(*q*). A spatially dependent cutoff time, *t*_*c*_(*q*, *R*), is not consistent with the assumption of a single coherent scattered wave, but may be amenable to a modal treatment. It is critical for the feasibility of this approach to identify the number of modes that are required, but we have not been able to do this from the one-dimensional simulation. The development of such a modal reconstruction method is beyond the scope of this work, but we hope that this discussion is helpful for the reader to appreciate the outstanding issues around reconstruction of an exploding molecule. We note also that if there are spatially dependent effects in ionization processes, due to the charging of the molecule, these would need to be handled by a similar modal treatment.

An alternative proposal for handling a Coulomb explosion is to use a tamper layer of water or buffer to cover the molecule (Hau-Riege *et al.*, 2007 ▸). The idea is that the tamper layer undergoes most of the expansion, leaving the molecule intact for the duration of the pulse. This assumes that the molecule is evenly coated with a water layer or sits centrally in a small droplet. It has been concluded from simulation that a 40 Å layer of water provides an effective tamper (Hau-Riege *et al.*, 2007 ▸). The buffer layer increases noise and background, as the positions of most buffer molecules are uncorrelated between measurements. The increase in noise from water scattering may be preferable to the explosion of a bare molecule, as a modal reconstruction of the expansion would not be needed. Noise will be suppressed with many measurements merged into a three-dimensional intensity. The extra noise is not expected to prevent the determination of molecular orientations, because it is still of similar order to the shot noise from diffraction of the bare molecule. We are not aware of a lower limit on signal-to-noise for Bayesian orientation methods, so long as a sufficient number of measurements can be obtained. Indeed, a two-dimensional orientation experiment succeeded with an average of only 2.5 photons per frame (Philipp *et al.*, 2012 ▸), which is extremely low. Some of the buffer molecules may bond to the target molecule’s surface with preferential position and orientation, producing diffraction that is correlated between independent measurements. These molecules may be reconstructed as lower-resolution surface features. The technical challenge of producing tamper layers of a controlled appropriate thickness remains outstanding.

## 5. Discussion   

The results presented here on damage noise have implications for the feasibility of determining the assembly of the three-dimensional diffraction volume from the ensemble of noisy two-dimensional measurements. The data-assembly algorithms use information common to different diffraction measurements to resolve unknown information about mol­ecular orientation. Predicting the level of damage noise in individual two-dimensional diffraction measurements is a first step towards understanding how damage affects these algorithms. The prediction that SNR_*D*_ is greater than 1 even for longer pulse durations (> 20 fs) is a preliminary indication that damage noise will not prevent data assembly under conditions currently available experimentally. This is because the contribution to the diffraction from the average molecular structure is greater than the shot-to-shot fluctuations of the diffraction, and it is the contribution from the averaged structure that is used to resolve the problem of unknown molecular orientations. That SNR_*ND*_(*q*) is lower than SNR_*D*_(*q*) by more than an order of magnitude (see Fig. 4 ▸) shows that shot noise dominates damage noise. This can be viewed positively because data-assembly algorithms can already cope with very low shot noise levels when assisted by *a priori* knowledge about the shot noise statistics (Loh & Elser, 2009 ▸; Fung *et al.*, 2009 ▸). However, shot noise applies per pixel and is well understood to be a Poisson process, whereas damage noise consists of fluctuations in the width of a speckle and the underlying distribution is hard to predict analytically. Detailed studies of the effects of damage on the performance of data-assembly algorithms are still required.

Since damage has been measured in nanocrystallography experiments, it is worth drawing a distinction between damage in crystals and in single molecules. In a crystal, damage ionizes and displaces ions differently in each unit cell, so that the diffraction contains an average over many different damage scenarios. For a single molecule, there is only one damage scenario per measurement and hence we expect a bigger standard deviation of diffraction of single molecules than of nanocrystals. Additionally, nanocrystals are much larger than single molecules, so that the rate at which electrons are trapped is different and the time it takes for a photoelectron to escape is longer. The water that surrounds a nanocrystal injected *via* a liquid jet (DePonte *et al.*, 2008 ▸) also contributes to the damage in the form of additional photoelectrons and secondary electrons. It is proposed to inject single molecules *via* aerosol injection (Bogan *et al.*, 2010 ▸), so that they are surrounded by vacuum, because the background water scattering from a liquid jet would dominate the diffraction from the molecule. For these reasons, damage experiments on single molecules, independent of those on crystals, are needed to draw conclusions for single-molecule imaging.

At X-ray energies approaching 10 keV, Compton scattering becomes another significant source of background scattering (Slowik *et al.*, 2014 ▸). The background is predicted to depend on the magnitude of *q*, and would increase the noise level σ_*N*_ by adding to the right-hand side of equation (8). It has been predicted that, for the beam intensities currently available at hard X-ray energies, the Compton background only becomes significant at resolutions greater than 2 Å (Slowik *et al.*, 2014 ▸). Hence, Compton scattering is not expected to influence the results presented here significantly.

## 6. Conclusion   

We have analysed shot-to-shot damage noise fluctuations for single-molecule diffraction. For spatially uncorrelated damage processes, our simulations show a damage gating effect for the average diffraction contrast, whereby the amplitude of the contrast increases with pulse time until a resolution-dependent cutoff time determined by damage. In our simulations, the damage noise introduced by uncorrelated damage processes is much less than shot noise, which provides a preliminary indication that there are favourable prospects for resolving molecular orientations to assemble in a three-dimensional diffraction volume in the presence of damage with data from current facilities. We have also proposed a statistical measure of damage that could be applied experimentally to provide valuable feedback for modelling XFEL damage to single biological molecules. The expansion of the molecule remains an outstanding issue that could potentially be addressed by a tamper layer or by a modal analysis of structural correlations, as is done in other dynamic coherent imaging applications. While both these solutions present a formidable challenge, our preliminary analysis suggests that both are deserving of further investigation.

## Appendix A Description of the rate-equations model

We use a damage model based on a rate-equations model (Hau-Riege *et al.*, 2004 ▸), which is extended to include ion diffusion using the methods from a non-local thermal equilibrium plasma model (Scott, 2001 ▸; Caleman *et al.*, 2011 ▸). The rates of photoionization were taken from Henke *et al.* (1993 ▸), rates of Auger decay were taken from McGuire (1969 ▸) and atomic energy levels were taken from Bearden & Burr (1967 ▸). Secondary impact ionization rates were taken from Bell *et al.* (1983 ▸) and Lennon *et al.* (1988 ▸). Ejected electrons are assumed to be trapped if their kinetic energy exceeds the trapping energy of the ionized molecule (Hau-Riege *et al.*, 2004 ▸). We assume a spherical geometry for this calculation, and this is the only place where geometry is included in the calculation. Both photoelectrons and some of the Auger electrons have sufficient energy to escape at early times. All of the trapped electrons are assumed to thermalize on a sub-femtosecond time scale, so that the energy distribution is Maxwell–Boltzmann, but the mean temperature changes with time. We include all ionization states of each element, and the electron orbitals for each ionization state were modelled using Slater-type orbitals (Slater, 1930 ▸).

There are some minor differences between our model and the published models on which it is based. We include all the shells for sulfur [in the work by Hau-Riege *et al.* (2004 ▸) it was restricted to eight electrons], and this introduces high-energy Auger electrons that are able to escape the molecule under the same conditions as the photoelectrons. We do not consider ionization due to potential lowering, as is done in the work by Scott (2001 ▸). We also omit the expansion of the molecule under electrostatic forces in order to focus on the spatially uncorrelated motion that is implicated in the self-gating pulse effect. The expansion of a protein molecule has been predicted to affect atoms less than one tenth of the molecule’s radius from the surface (Hau-Riege *et al.*, 2004 ▸). These atoms can move several ångströms during interaction with the pulse, which will greatly diminish their contribution to the diffraction contrast. The rest of the atoms are only weakly affected by expansion because the trapped electrons effectively neutralize the core, for which we would expect better agreement with the theory presented here.

The simulation of the Coulomb explosion as the movement of one-dimensional layers is based on that of Hau-Riege *et al.* (2004 ▸). We briefly summarize the calculation here and refer the reader to Hau-Riege *et al.* (2004 ▸) for further details. The trapped electron density is solved at each time step as a two-point boundary value problem, using a differential equation that combines the Coulomb forces from the ions and trapped electrons and the radiation pressure of the trapped electron gas. The distribution of trapped electrons and ions is then used to calculate the radial acceleration of each layer, using an artificial viscosity to avoid shock waves arising from numerical instability. The equations were solved with a time step of 10^−4^ fs, which is within the limits described by Hau-Riege *et al.* (2004 ▸) for numerical stability.

## Appendix B Derivation of equation (4)

The intensity of a measurement can be written as

where the definitions of all terms are given in the main text. We can expand the cosine term as

We can further expand the terms that depend upon the displacement as

The ensemble averages of individual cosine and sine terms over different random displacements are

and

We assume that ionization and atomic motion are statistically independent, so that

We assume that the ionization of different atoms is statistically independent, so that

if . We assume that all atoms of the same element are statistically equivalent, so that averages of *f*_*i*_(*q*, *t*) and  are independent of *i*. Combining the above results we obtain

Substituting equation (26) into equation (19) leads to equation (4), using the definitions of *A*(*q*) and *B*(*q*) in equations (5) and (6), respectively.

## Appendix C Derivation of the variance of *A_i_*(*q*): equation (9)

The standard deviation of the sum of *A_i_*(*q*) terms in equation (1), denoted by σ_*A*_(*q*), is given by

with

Equation (27) is scaled with the number of atoms to give the contribution per atom. We ignore the *i* dependence when writing σ_*A*_(*q*) because we assume all atoms of the same element are equivalent. Using the assumption that ionization on different atoms is statistically independent, we can write

Therefore,

## Appendix D Derivation of the variance of *B_ij_*(*q*): equation (10)

The term σ_*B*_(*q*) gauges the magnitude of the damage noise fluctuations per atom due to the second term on the right-hand side of equation (1). Its square is related to the difference between the variance of the second term on the right-hand side of equation (1) and that of the second term on the right-hand side of equation (4), which is given as follows

where σ_*S*_(*q*) is defined to be the standard deviation of the second term on the right-hand side of equation (1) and is given by

The second term on the right-hand side of equation (19) contains terms with the form

where we have defined

and

Using equation (23) we can show that

and thus write

We evaluate  as a first step to calculating the standard deviation

Going from the first to the second line of equation (38), we have used the assumption that the positions of the atoms are random, so that

and, in the last line of equation (38), we have

To evaluate equation (38), we start by evaluating  as follows

Writing , we can write

The term  is given by

The joint probability function is

Assume that . We then assume that the conditional probability is the probability of taking a random walk from position  at time  to position  at time *t* and takes the form

where  is given by the integral of the diffusion coefficient as a function of time

The term *N*_D_ is the number of dimensions, which we will take to be 1 because we are only interested in diffusion in the direction of the scattering vector. The diffusion coefficient is given by

where *k*_B_ is Boltzmann’s constant, *T*(*t*) is the ion temperature, *m* is the ion mass and ν(*t*) is the collision frequency. To evaluate equation (43), we first write each cosine term as a sum of exponentials

We then solve two integrals of the form

The first integral is over , with *a* =  and *b* = . The argument of the resulting exponent is

The second integral over  has

The final summation over *m*, *n* = 0, 1 gives the following result for

The corresponding sine integral evaluates to

Adding the cosine and sine integrals, we get

To complete the evaluation of equation (38), we still need to evaluate  which is given by

This equation can be written in the form

Using equations (52) and (53) we can write this as

We can write the time integrals as

Using the property that , equation (58) can also be written as

Using equations (38) and (59) and the fact that , we can calculate the standard deviation of *B_ij_* [denoted ] to be

We have now reached a point where we can evaluate , given by equation (32). The averages of the terms  are zero unless *i*, *j* = *r*, *s*, because the averages over the positions **R** equal zero. Therefore,

Using this result in equation (31), we obtain the following result:

Assuming that *N* is large, the term 1/*N* can be ignored.

## Appendix E Results for multiple elements

Following the formulation of Quiney & Nugent (2011 ▸), we can generalize the key results of this paper to multiple elements. We have used the versions for multiple elements in our simulation study. We use the notation *f_Z_*(*q*, *t*) to denote the scattering factor of the element with atomic number *Z*. We can then rewrite equations (5) and (6) as weighted averages over all atomic species. Using *P_Z_* to denote the fraction of atoms of element *Z*, equations (5) and (6) become

and

where

The term  denotes the root mean square (r.m.s.) displacement of an ion of element *Z* as a function of time. The weighted averages of  and  over multiple elements are given by

and
